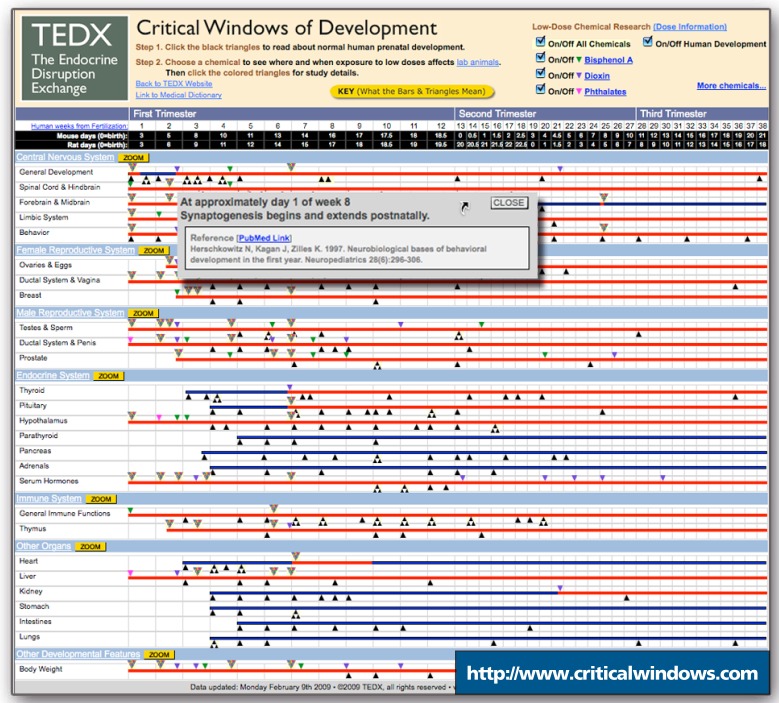# ENDOCRINE DISRUPTION: Developmental Picture Window

**DOI:** 10.1289/ehp.117-a101

**Published:** 2009-03

**Authors:** Julia R. Barrett

The concept of endocrine disruption emerged in the early 1990s with the observation that natural and industrial compounds were interfering with sex hormone signaling, thereby adversely affecting male and female reproductive health. Since then, many endocrine-disrupting chemicals (EDCs) have been identified, and the field now comprises thousands of studies encompassing virtually every system in the body. On 10 February 2009, the Paonia, Colorado–based nonprofit group The Endocrine Disruption Exchange (TEDX) unveiled the Critical Windows of Development timeline (http://www.criticalwindows.com). The timeline provides a snapshot of the state of the science of when organs and systems develop, when they are vulnerable to particular EDC exposures, and what effects have been observed.

Endocrine disruption is not confined to the reproductive system because virtually every system in the body is hormonally responsive. The effects can differ depending on when exposure occurred. For example, according to the timeline, prenatal exposure in mice to bisphenol A (BPA) at gestational days 11.5–18.5 is associated with abnormal fetal egg cell development whereas exposure at gestational days 15–18 is associated with structural changes to the vagina.

Theo Colborn, president of TEDX, first conceptualized condensing the body of EDC literature into a user-friendly graphic format. Despite Colborn’s optimism concerning the timeline’s feasibility, it took several years to figure out how to present the data visually. “We had the data here, and we had everything in boxes, but displaying it in the picture we wanted to create was complex,” says Colborn. An early attempt involved an extraordinary expanse of butcher paper; a collaboration with Carol Kwiatkowski, now executive director of TEDX, helped bring Colborn’s vision to fruition. “As far as we know, there’s nothing out there like it,” says Kwiatkowski, who organized and then funneled the research into a database before finding the web developer who could translate it into the desired visual display.

The display comprises a series of horizontal bars, each depicting a specific system or organ for the full 38 weeks of human pregnancy. Corresponding time points in rodent development are indicated along the top of the screen. Tick marks along the bars indicate studies done at specific time points corresponding to points in normal human development. Another series of tick marks indicates EDC studies performed in the laboratory. Clicking on a mark brings up a concise summary of the study details with a link to the PubMed record.

All chemical studies notated on the timeline must be original research using rodent or human cells or tissues. Exposures of parts per million or less to an EDC must have occurred during a time point equivalent to some point in human pre-natal development. “We wanted to keep it within a range [representing the point] where we know from the literature on ambient exposures and monitoring studies that humans might be exposed,” says Colborn. The timeline is currently populated with all existing BPA studies that meet the criteria for inclusion; staff are still plotting dioxin and phthalate studies. New studies will be inserted upon publication, and more chemicals will be incorporated into the timeline in the coming year.

The timeline, which is free to all users, fills several needs at once. “One of the needs that has become apparent over the years has been just a basic reference for normal development in both humans and rodent models,” says Kris Thayer, a health science administrator at the NIEHS. To begin to grasp how various systems are affected by endocrine disruption, it is necessary to know when those systems are developing. “What Theo has done is to develop a graphic tool—it’s very visual, very interactive, and very well referenced—so you can easily see everything in one place,” says Thayer.

Jerry Heindel, a scientific program administrator at the NIEHS, says the time-line could be useful in determining areas where research is needed and how to plan new studies. “Even a cursory examination of the tick marks that show when experiments have been done provides a nice overview of the timing of studies throughout development,” he says. “This information can then be used to refine the timing, doses, and end points of future studies.”

Colborn also hopes the timeline will inspire scientists to take a broader view of endocrine disruption and possibly promote collaboration across systems. Researchers tend to focus on one system or organ at a time, she says, and as a result a lot of information that could be gleaned from carefully controlled experiments is never gathered.

“I think the timeline will be very useful for researchers [from many disciplines], especially as people cut across different biological systems,” says Thayer. “The information on the timeline is contained in reference books and research journals, but it’s very handy to have it all in one place.”

## Figures and Tables

**Figure:**